# A qualitative evaluation of the reflective fostering programme – examining foster and kinship carers’ experiences, practical application, and perceived impact

**DOI:** 10.1177/13591045251321032

**Published:** 2025-02-19

**Authors:** Thando Katangwe-Chigamba, Jamie Murdoch, Karen Irvine, Sheila Redfern, Nick Midgley

**Affiliations:** 1 Norwich Clinical Trials Unit, Norwich Medical school, 6106 University of East Anglia, UK; 2 School of Life Course and Population Sciences, 4616 Kings College London, UK; 3 Centre for Health Services and Clinical Research, 3769 University of Hertfordshire, UK; 4 4785 Family Trauma Department, Anna Freud National Centre for Children and Families, UK; 5 Research Department of Clinical, Educational and Health Psychology, University College London, UK

**Keywords:** Mentalization, foster carer, reflective functioning, self-regulation and self-mentalizing

## Abstract

**Background:**

Mentalization is the ability to think about and interpret behaviours of both self and others in terms of thoughts and feelings. Caregiver’s capacity to mentalize can enhance the quality of parent-child relationships. The UK Reflective Fostering Programme (RFP) has been developed to enhance foster and kinship carers’ self-regulation and self-mentalizing.

**Aim:**

To understand carers’ experiences, practical application, and perceived impact of the RFP.

**Methods:**

A qualitative evaluation of the RFP using in depth interviews with twenty-four carers. Interviews were audio-recorded, transcribed verbatim and analysed thematically.

**Results:**

Three key themes were identified: (1) ‘*Me time’ – carer’s reflections on the programme and what makes it work*, highlights sharing of challenging experiences as important for practising mentalizing. (2) ‘*Stopping and thinking in the moment’ - carer’s understanding of programme concepts* explores carer's conceptualizations of mentalization, revealing some male carers described challenges regarding sharing personal experiences in a group and the practical application of mentalization. (3) *Practical application and perceived impact of the programme* suggests successful enactment of learning from the programme, resulting in enhanced capacity to cope with stress, prevention of outbursts and better communication with children in their care.

**Conclusions:**

Our findings suggest the programme can successfully facilitate carer’s use of self-mentalizing, leading to greater self-regulation and capacity to support children in their care. Future research should explore experiences of male and kinship carers to inform tailoring.

## Plain language summary

Mentalization is the ability to think about what we or others are thinking or feeling. Research shows that when caregivers mentalize, they are better able to control their own feelings and reactions, which can in turn improve relationships with their children. In the UK, the Reflective Fostering Programme (RFP), a group-based programme, has been developed to improve foster and kinship carers’ ability to mentalize. To understand experiences of carers’ attending the programme, we interviewed twenty-four carers who had attended the programme. We wanted to know whether they applied what they had learnt and if it had helped their relationships with their children. Carers told us that hearing and sharing stories of challenging experiences during the sessions helped them to practise mentalizing and understand why they or their children behave or react in certain ways. Most carers understood mentalization as the ability to think about what they or others are thinking or feeling. However, some male and kinship carers felt mentalizing is unhelpful because one cannot know for sure what other people are thinking or feeling. Most carers said that mentalizing had helped them to stop and think about what others are thinking or feeling, before reacting. By doing this some participants reported having less heated arguments with their children and others said mentalizing had helped them to cope better with stressful situations and had helped improved communication with their children. Overall, our findings suggest that by attending the programme, carers were better able to behave in ways that can improve relationships with their children. However, more work needs to be done to understand whether male or kinship carers experience the programme differently, and whether changes need to be made to help them benefit more from the programme.

## Background

Mentalization is the ability to think about and interpret behaviours of both self and others in terms of mental states, thoughts and feelings (Fonagy & Luyten, 2009; Fonagy et al., 2002; Fonagy & Target, 2006; Sharp & Fonagy, 2008a). Children in foster care have often experienced early childhood trauma, such as emotional neglect and maltreatment (Clausen et al., 1998). Such experiences, augmented by frequent changes of caregivers and their living environment, often create challenges in forming secure attachments leading to adverse effects on their emotional well-being and development.

According to attachment theory (Ainsworth et al., 1979), sensitive caregiving, involving accurately perceiving and interpreting a child’s signals followed by prompt and appropriate responding, develops an internal working model of the primary caregiver as a ‘secure base’ from which the child can explore the environment, and feel assured that comfort and protection will be available when required. In contrast, insensitive caregiving, involving repeated interactions where a caregiver rejects or fails to meet a child’s cues for proximity/emotional support, is associated with internal working models of the caregiver as unavailable or unreliable, and insecure attachment patterns as compensatory strategies to deal with the resulting relational stress (Main & Solomon, 1986; Main & Solomon, 1990).

There is, therefore, a strong evidence base emphasising the vital role that caregiver’s capacity to mentalize (operationalised as ‘reflective functioning’) plays in enhancing the quality of the parent-child attachment relationship (De Wolff & van Ijzendoorn, 1997; Ensink, Berthelot, et al., 2014; Sharp & Fonagy, 2008b; Zeegers et al., 2017). Caregivers demonstrating a higher level of reflective functioning have been associated with better communication, a higher tolerance to children’s distress (Rutherford et al., 2013), the ability to manage stressful situations (Borelli et al., 2016), and positive parenting skills (Rostad & Whitaker, 2016). Evidence also shows that higher caregivers’ reflective functioning promotes secure attachments, greater self-esteem, and social competence in children (Berube-Beaulieu E et al., 2016; Borelli et al., 2012).

The established links between caregiver mentalizing and sensitive caregiving (Ensink, Berthelot, et al., 2014; Ensink et al., 2014b; Fonagy & Luyten, 2009), and between child attachment security and subsequent positive social, emotional, and psychological outcomes, have led to mentalization-based training programmes being recommended for foster carers (Howard & Miriam, 2018; Moore T G et al., 2017). Such programmes aim to improve parental capacity to provide sensitive and responsive caregiving, with the goal of improving child attachment patterns.

In the UK, the Reflective Fostering Programme (RFP), a group-based psychoeducational intervention aiming to support foster and kinship carers of children aged 4–11 (Redfern et al., 2018), has been developed following calls (Adkins et al., 2018; National Institute of Health and Care Excellence, 2013; National Institute of Health and Care Excellence, 2021) to improve outcomes for children in care. The programme draws on attachment and mentalization research (Cooper & Redfern, 2016; Laranjo et al., 2010; Meins et al., 2003; Slade, 2005), but primarily focuses on developing carers’ self-regulation and self-mentalizing in relation to children in their care. More practically, the programme aims to provide carers with tools that represent the principles of reflective caregiving in a shortened, highly applicable form.

The RFP shares a number of features with other fostering programmes, including a focus on understanding the impact of trauma and child development, and an intention to support positive carer-child relationships; however it differs from other widely implemented UK based programmes such as Fostering Changes (Briskman et al., 2012), which draw on cognitive-behavioural models and focus primarily on providing practical coping skills for carers. The RFP also differs from other attachment-based programmes such as the Attachment and Biobehavioural Catch-up (ABC) which targets caregivers of infants and young children (6–48 months) and primarily focus on supporting appropriate interpretation of child cues in parent-child interactions (Dozier et al., 2014).

The RFP has been piloted and feasibility tested in two single arm, non-randomised studies, with findings indicating positive changes in carer stress levels, child’s emotional and behavioural well-being and capacity for emotion regulation (Midgley et al., 2019, 2021b). A small qualitative evaluation embedded in the feasibility study indicated the programme to be acceptable and relevant to foster and kinship carers’ needs. The RFP is currently being evaluated for effectiveness and cost-effectiveness in a large-scale randomised controlled trial , the Reflective Fostering Study (Midgley et al., 2021a). Across the trial, an embedded mixed methods process evaluation aimed to describe how the RFP was delivered, assess intervention fidelity, understand how contextual factors shaped intervention delivery, and provide explanations for the observed effects of main trial findings. This paper, reporting one of the process evaluation objectives, aims to understand foster and kinship carers’ experiences, practical application, and perceived impact of the RFP in their relationships with children in their care.

## Methods

### Study design

A qualitative process evaluation of the RFP (Midgley et al., 2021a), using in depth interviews with carers. This study was part of the Reflective Fostering Study, a pragmatic, randomised controlled trial, with a nested process evaluation and economic evaluation (52).

### Study setting

The Reflective Fostering Study took place in local authority (LA) fostering teams and independent fostering agencies (IFA) across the UK. Four sites participating in the trial were purposively selected to participate in the nested process evaluation: Lancashire County Council (Northwest England, 777 children, 1082 carers), North Tyneside Council (Northeastern England, 117 children, 146 carers) and Fostering People (IFA providing services across the UK, 460 children, 350 carers) and Anna Freud Centre (Mental health charity for children and families, delivered the programme to carers from a mixture of fostering agencies).

### The reflective fostering programme

The RFP is a 10-session programme offered to a group of 6–10 foster carers over 12–14 weeks. Originally developed to be delivered face-to-face, the programme was adapted during the COVID-19 lockdowns in the UK to be delivered online (Redfern et al., 2023). Groups are co-facilitated by a social worker and a foster carer, following a three-half day training. The programme content consists of psychoeducation on themes including ‘network of support’ and ‘impact of trauma’, combined with opportunities for carers to share experiences they are having with the children in their care, with an opportunity to develop their mentalizing capacities. The programme uses several practical tools to help carers keep in mind and practise mentalizing self and other, including:

a) The Carer Map: a tool designed to help carers identify important influences on their parenting including, the impact of past family history, current influences and states of mind, and to see how these influence their caregiving.
b) The Carer APP (**A** ttention and Curiosity; **P** erspective Taking; **P** rovide Empathy): a tool to help carers learn how to mentalize their foster child, that is, to be curious about and reflect on the meaning behind their behaviour.
c) The Emotional Thermometer: a tool designed to help carers monitor their own emotional arousal to regain mentalizing capacity.
d) The Two Hands Approach: a tool that helps carers understand the balance between action and reflection in interactions around discipline. “Two hands” refers to both dealing with or directly responding to a difficult behaviour and understanding what led to it (the mentalizing/reflective process).

Each session, lasting 2–3 hours, begins and ends with a Mind Check to practice tuning into current state of mind, and includes some psychoeducation, theme of the day, exercises and discussion points. More details about the programmes’ model, structure and content have been reported elsewhere (Midgley et al., 2021a; Redfern et al., 2018).

### Participants and sampling

Twenty-four foster or kinship carers (also known as ‘connected carers') participating in the intervention arm of the Reflective Fostering Study. In each of the four process evaluation sites, we initially intended to purposively select carers to achieve a maximum variation of years of experience as a carer, ethnicity, gender and whether a kinship or foster carer. However, due to low numbers of kinship and male carers willing to participate in the process evaluation, participants were primarily conveniently sampled. Carers demographics are presented in Table 1. Closely reflecting demographic of foster carers in the UK (Office for National Statistics (ONS), 2021, Ofstead, 2023), most participants were female (87.5%), foster carers (91.7%), over the age of 50 (83.4%) and from a White ethnic background (79.1%). Fostering experiences ranged from 0-20 years.

**Table 1.** Carers Demographics.

Characteristic *n* = 24	*n* (%)
Age	
30–39	2 (8.3%)
40–49	2 (8.3%)
50–59	13 (54.2%)
60–79	6 (25%)
Missing data	1 (4.2%)
Gender	
M	3 (12.5%)
F	21 (87.5%)
Ethnicity	
White british	19 (79.1%)
Black caribbean/British	3 (12.5%)
White Irish	1 (4.2%)
White and caribbean	1 (4.2%)
Type of carer	
Foster	22 (91.7%)
Connected	2 (8.3%)
Fostering experience (Yrs)	
0–9	15 (62.5%)
10–19	7 (29.2%)
20–29	2 (8.3%)

### Data collection

Semi-structured interviews conducted virtually via Teams or over the telephone. Interviews were audio recorded and were facilitated by a topic guide developed by the research team (see supplementary file 1). Topics covered included carers’ relationships with the child(ren) in their current placement; experiences of participating in the programme; views and perceptions of programmes’ structure, activities, concepts and tools. Interviews lasted between 45-60 minutes and were conducted between 4–12 months from baseline. Separate written, informed consent to participate in the qualitative study was obtained from all participants.

### Analysis

Interviews were transcribed verbatim and anonymised transcripts uploaded onto NVivo for analysis. Data were initially analysed thematically (Joy et al., 2023) using the following steps: (1) Familiarisation; (2) Inductive Coding; (3) Theme generation; (4) Theme development; and (5) Refining, defining and naming themes (Braun and Clarke, 2013). Theme development was also guided by the research questions and the RFP logic model (Midgley et al., 2021a; Redfern et al., 2018), where for example, codes relating to carers’ application of key principles of reflective caregiving were mapped against the respective programme tools (e.g., Carer MAP) to provide an understanding of whether and how carers applied different types of tools/principles of the Programme. Finally, the themes were refined through discussions and feedback from members of the research and intervention development team.

## Results

**Figure 1.**
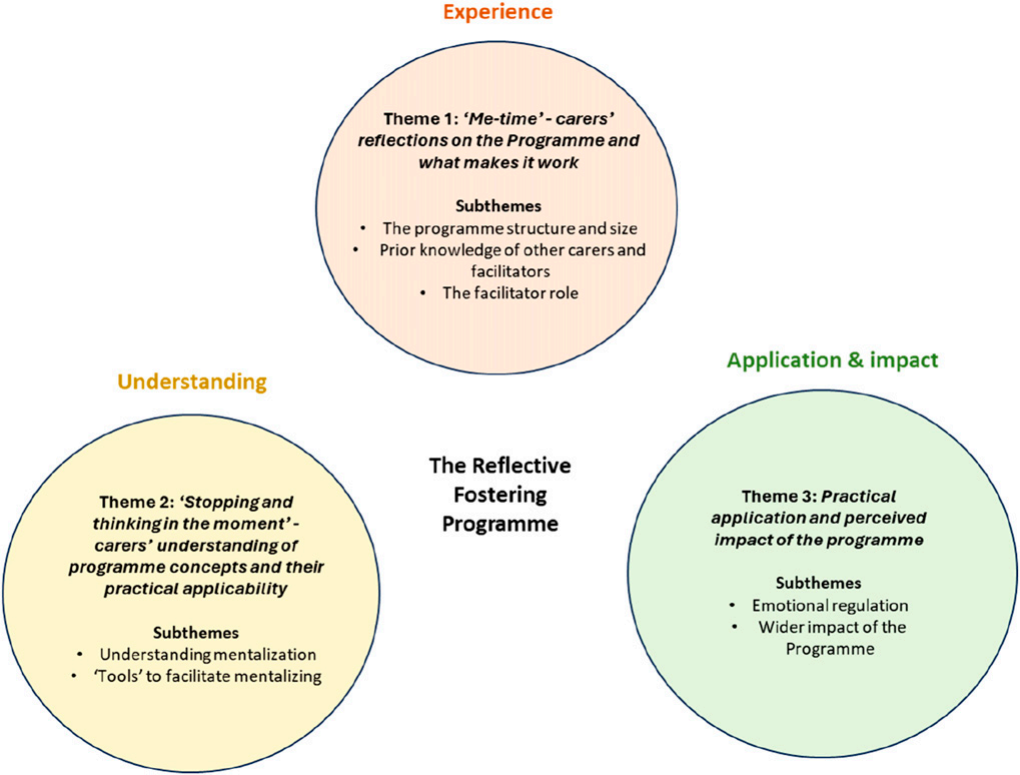
Themes and subthemes.

Three key themes were identified from the analysis (see Figure 1). The section below provides a narrative of each theme with illustrative quotes. Fuller and additional illustrative quotes are presented in Table 2 and referenced within the text.

**Table 2.** Themes, Subthemes and Illustrative Quotes.

Theme	Subthemes	Illustrative quotes	
1. ‘Me-time’ - carers’ reflections on the programme and what makes it work	1.1 The programme structure and size	A	When you hear other carers and they’re going through a similar thing, and you think, “oh, they actually do make sure they get time for themselves. I’m going to make sure I get time for myself. And I shouldn’t feel guilty about getting time for myself, and that’s really helped as well, because they were all like, “no, you need to, to keep your cup flowing,” and I was like, “yes, you’re right.” it just kind of cleared everything. [C12, age 35, female, white, foster, 1 Yrs. Exp .]
		B	Talking to the other foster carers and stuff, you realise you’re actually not the only one that’s struggling. You’re not the only one that sometimes bangs your head against a brick wall. And it is OK not to feel good, and it is OK to step back and think ‘hang on, I need a bit of time to think about this, what I’m going to say or what I’m going to do. [C6, age 64, female, white, foster, 11 Yrs. 2 mo. Exp.]
			I was very critical of myself when I started my fostering journey. I Was very lacking a lot in confidence. I Think the reflective fostering programme really helped me to step back and appreciate that I was doing my best and appreciate that she [the child] was making progress [C4, age 56, female, white, foster, 0yrs 9 months exp.]
		C	I just thought there weren’t people there with different ideas...It was very narrow. Because I thought it was going to be about 12 people. I think most weeks we had probably four plus two [facilitators]. I’m not criticising it; I thought the course was lacking in some sort of decent debate [C16, age 64, male, white, 6yr 6mo exp.]
	1.2 prior knowledge of other carers and facilitators	D	I would probably find it easier [if I didn’t know everyone]. Because of the issues we face with [childs name], I’m very aware that all foster carers know each other, all have social events together. So, I would always be careful what I said about him because I would hate to be in a social setting with [Child’s name] and have someone there who’s heard not nice stuff, to think ‘oh hell, he’s here’ [C7, age 52, female, white, 6 yrs 3 mo. Exp.]
	1.3 The facilitator role	E	There was no “oh, I can’t say that because she’s a supervising social worker”. The [facilitators] talked quite freely about how they felt if they were having situations. Even the supervising social worker, she was having some situations in her own homelife, and it just made it so real, and everybody’s got these situations, and everybody comes to points where you can’t cope, and you question yourself [C3, age 58, female, white, foster, 10 Yrs. 2 mo. Exp.]
		F	I revealed to everybody about [that) my child is violent towards me…people were sympathetic, but it was just opened up to the group, “could anybody suggest anything that might help?” and nobody really had any ideas. And I was quite upset because it was already a difficult time and I felt that I’d really done a big thing in putting that out there and exposing that to other people in that there wasn’t any support. It was hard [C4, age 56, female, white, foster, 0yrs 9 mo. Exp.]
			So, nobody told me that what I was saying was wrong, but they then just very smoothly and gently moved on to something else without making any comment. I know what that means, “you’re barking up the wrong tree, you’re overreacting. Let’s move on to somebody who’s got a more sane point of view.” it was subtle, but I felt put down. And I’m probably oversensitive, almost certainly, but this is an incredibly sensitive area **[**C13, age 69, female, white, connected, 3 yrs 8 mo. Exp.]
		G	I think the social worker would give me confidence that it was a professional approved conversation or material, I suppose, rather than if it was just run by carers [C4, age 56, female, white, 0 Yrs. 9 mo. Exp.]
			I thought that was very good to be honest with you, I think if it was just, I’m not saying it wouldn’t have been good, but with [social work professional’s’ name] it brought about a bit more of an understanding if that makes sense. So she was, if we said something like I don’t know, “oh my social worker doesn’t get back to me” etc., we’d know that they’ve got a lot of work on, we understand that. It was nice because [social work professional’s’ name] wouldn’t be defending them but she’d speak from experience and that helps [C24, age 48, female, Black,10yrs 3 mo. Exp.]
		H	I think it’s because social workers, yeah, they’re really involved and they know their cases inside and out, but unless you’re living it and you’re doing it and you’re experiencing it with that with a child, it’s quite difficult to imagine and appreciate and to fully comprehend what it’s like [C2, age 44, female, white, 3yrs 11mo exp.]
		I	More social workers should attend [the programme]… and if they don’t do it with us then I think a video should be done […] I’ll tell you why because people need to understand what we go through […], even if it’s a little five to 10 minute video then for that moment they might then have some empathy on us and think, “oh my gosh you know the next time I phone up [carer’s name] and I say can you just do this and do that, I’m going to think you know what I’ve just loaded her with a lot of stuff, offloaded it off myself, but how is she going to deal with this. And what might she need more to ensure that she can keep this placement”. Because I’m saying it to you now in plain black and white, if you do not fill up a carer’s cup they will close that, throw that water away [C24, age 48, female, Black,10yrs 3mo exp.]
		J	That’s really important for these courses to have someone when we’re all a bit silent thinking, “oh I’m not sure what she means, what sort of examples is she asking for?” and then she says, “okay, here’s an example, what happened to me this week and how I used this, or how I could have done better with this approach”. And then actually then you go, “okay, alright, I get it now, right, okay, this is, you know”, and it allowed us to… , so I think there [C17, age 52, male, white, foster, 7 Yrs. Exp.]
Theme 2: ‘Stopping and thinking in the moment’ - carers’ understanding of programme concepts and their practical applicability	2.2 understanding mentalization	K	So, we’ve done reflection at the end of the day with him and also then once he’s in bed, I’ve thought through different things that have happened during the day and thought, “I wonder if …” it just helps you think that next time if that happens, “I wonder if I can try this or try that?” [C15, age 51, female, white, 0yr 11mo. Exp.]
		L	Yeah, it [mentalization] definitely does work. I mean I think I’m quite a reflective person anyway. I would mostly do it in bed at night, I’d toss and turn things over, remind and think about what I could’ve done differently whereas this [the reflective fostering programme] has made me stop and think in the midst of things […] to try and think about things as it’s actually happening, this has really helped me to do that [C4, age 56, female, white, foster, 0yrs 9 months exp.]
		M	They do lots of work on where the child’s at and how they feel...the side I didn’t really do much on before was including myself in it. I wouldn’t think about myself in a situation where I was trying to sort out an issue that was going on with a child I was looking after. I wouldn’t stop to think, “how does this make you feel? Are you reacting the right way? Is this going to work out? Is it going to benefit? [C14, age 58, female, white, 12yr 8mo exp.]
		N	I hated that word [mentalizing]. I hated that with a passion. Because to me it’s thinking about thinking, and you can’t do that. I Can look at your body language and all that, but I’ve no idea what you’re thinking now. I just think it’s the wrong – I just thought all the phrases they use were wrong. You can sit in a room with 50 people and think “I know what he’s thinking,” but you don’t, do you? [C16, age 64, male, white, 6yr 6mo exp.]
	2.2 ‘tools’ to facilitate mentalizing	O	They’re great theories, but actually the practice is what do you do. “When you feel you’re going to snap, what do you do? Do you leave the room? Do you stay there and keep it under control? What is the best thing to do, and what is the next best thing to do? [C13, age 69, female, white, connected, 3yr 8mo exp.]
			My view is you don’t get out bed thinking, “oh, should I use that tool”. You get out of bed and you do what you do…but going back to the course itself, reflective fostering, you do reflect on what you’ve done, don’t you? So that in itself is the only term I think that’s probably genuine to the course. All the other things, again, are subjective and objective depending on your view [C16, age 64, male, white, 6yr 6mo exp.]
Theme 3: Practical application and perceived impact of the programme	3.1 emotional regulation	P	I am finding myself now, when I wake up in the morning, what kind of headspace am I in? The temperature. Am I cold? So I’m aware of how I’m feeling is going to impact if something happens [C2, age 44, female, white, 3yrs 11mo exp.]
			You’d empty your head first thing. It was a really good thing to do because there were a couple of weeks where I came, and I had lots of little things going on in my head. As I was saying them out to the person that I was talking to, I was writing them down at the same time, and it got them put down and they were out of my head then. Then you could concentrate on what was going on […] so I’ve used that more since the course because I realised really how well it works for me [C15, age 51, female, white, 0yr 11mo exp.]
		Q	It’s changed my approach entirely. I mean I’ve had children in here that have cut themselves and stuff like that, and I’ve just approached them softly. And one of them said to me one day, “why are you not shouting at me?” I said, “What’s the point of shouting? There’s no point shouting, it doesn’t get us anywhere does it?” I said, “You need help, and that’s what we’re here for, to help you, not to punish you [C1, age missing, female, white, 10yr 2mo exp.]
			She [the child] has these emotional outbursts […] We’ve avoided a lot of those outbursts really because she’s almost sometimes maybe waiting for you to say something, or challenge it. And I think by not challenging it, she’s gone away and relaxed and we’ve not taken it on if you like. So, it’s saved a lot more explosive outbursts than there could have been [C3, age 58, female, white, foster, 10 Yrs. 2 mo. Exp.]
			I don’t raise my voice as much, where I used to before, because it was like, I used to think, it’s embarrassing. We’re out and he’s doing this again. But now I’m thinking, no, I’ll switch off to it [C6, age 64, female, white foster, 11 Yrs. 2 mo. Exp.]
	3.2 mentalization of the self and other	R	Before, if there was an eruption in behaviour, I used to just go and give the discipline straight away. But now, I’ll make the point of calming him down first and stopping the whole situation before we discuss it. But discussing it could be the following day. It depends on how long it takes him to calm down. Because I found that if I didn’t stay calm, the punishment got worse [C6, age 64, female, white foster, 11 Yrs. 2 mo. Exp.]
			Is it the carer’s map […] I quite like [the carer’s map]that one. I find that for me and for the girl that I look after. So like food issues, I can understand that one. I can know that when I was younger, I didn’t get a lot of food. So I can empathise with the child I look after …… not having a lot of food. I can say to them, “I know how it feels because I didn’t always get all my meals every single day. That’s why I’ll make sure you will. That’s why I make sure we do [C14, age 58, female, white, 12yr 8mo exp.]
		S	So if I would get cross because my expectation of her is to be able to go off and do X, Y and Z and she doesn’t do it. But because I’d never analysed me or mentalized myself on it, it’s kind of escalating into a bit of a shouting match, but then when I could step back and go, “You’re getting yourself cross because she’s not doing the things you want her to do, why are you getting cross about it?” and I’d be like, “does it really matter in the grand scheme of things? Is it a fight worth fighting or do we let it go?“. [C14, age 58, female, white, 12yr 8mo exp.]
			I’d always just accepted that I’d had a great childhood and everything was rosy. That week where we stepped back and looked at our family [I] realised that everything wasn’t always rosy…it helped me to understand that’s just part of the journey that you got to learn how to parent, and particularly with a very challenging little girl. I needed to cut myself some slack and accept that she was progressing, and I must be doing it right [C4, age 56, female, white, foster, 0yrs 9 months exp.]
		T	The lad I’ve got, he’s got very complex needs and he gets very angered, but my approach to him now, from what I’ve learned, is approach him gently, put myself in his place and understand what he’s going through, and explain to him, “I understand how you’re feeling and I understand what you’re going through, and I’m here to help you,” and just get him to calm down. Yeah you do see a change in him…it does work [C1, age missing, female, white, 10yr 2mo exp.]
	3.3 wider impact of the reflective fostering programme	U	It has worked with the little girl. She’s so much better because I can understand her a bit more and understand the trauma she’s been through. And putting yourself on their level really works for me, and it works for the girl. I’ve also used it [with] my daughters. I use it with my husband. I just think, “right, let’s try this one on you now.” and he went “I haven’t got a reaction.” [C1, age missing, female, white, 10yr 2mo exp.]

### Theme: 1 ‘Me-time’ - carers’ reflections on the programme and what makes it work

The RFP’s emphasis placed on carers looking after, and mentalizing, themselves (not just the children) was highlighted as a unique aspect of the programme. Whilst for some, *looking after themselves* was equivalent to attending the programme itself, for others this meant taking time out to do activities for themselves without feeling guilty (Table 2, quote(s) A). About the decision to join the study and attend the programme, one carer explained:

Initially, I thought “What have I done? I’ve given such a commitment and I’m such a busy person.” But after the first couple of sessions, I found actually I feel like this is my time. [C3, 58, Female, White, Foster, 10 Yrs. Exp.]

The focus on carers is operationalised through the experiential aspect of the programme, whereby facilitators actively model and invite group members to think about the impact that caring for the children has on them, and to notice when they feel overwhelmed or unable to think calmly. Carers were largely positive about this aspect of the programme, highlighting several benefits: firstly, sharing personal experiences was thought to have facilitated shared learning, particularly where more experienced carers were giving accounts of how they have previously dealt with challenging behaviours. Secondly, hearing other’ stories helped to normalise experiences and reactions, thus combating feelings of isolation and guilt. For some newer carers, the shared understanding within the group, and the empathy and validation received from other carers and facilitators, resulted in them being less self-critical, more confident and appreciative of their own efforts and progress with the child(ren) in their care (Table 2, quote(s) B). Finally, some carers likened the experiential aspect of the programme to being in ‘therapy’, where they saw the sessions as a safe space to reflect on difficult experiences and be heard by others who could relate to their experiences.

It's sort of cathartic and felt as if you learnt a bit but actually you feel better at the end of the couple of hours. However you went in, however stressed …that’s why I call it support group yoga because it was two hours when you're thinking about yourself [C17, 52, Male, White, Foster, 7 Yrs. Exp.]

However, not all carers felt positively about sharing personal experiences. For example, one male carer described this activity as contrary to their approach to coping, which was to mainly focus on the positives in life and not dwell on negative experiences.

In terms of mindset, I block out any negativity, and sometimes the training sessions with some foster parents – if they’re going through a particularly bad time, were a struggle…it can be quite a negative environment and that can sometimes bring you down [C9, 34, Male, White, Foster, 2 Yrs. Exp.]

The sharing of personal experiences and challenges required openness and a level of vulnerability from the carers. Carers used terms such as ‘supportive’, ‘understanding’, ‘non-judgmental’ and ‘safe’ to describe the space which enabled them to openly share their experiences. Three key contextual features of the programme were highlighted to have facilitated (or hindered) building trust and feelings of ‘safety’ to share personal experiences:

#### The programme structure and size

Confidentiality assurances highlighted throughout the programme were an important feature that facilitated trust. In addition, the use of online breakout rooms, where carers did activities such as the Mind Check in pairs, also facilitated initial reflecting, without having to address the whole group. The length of the programme (10-sessions) was another element highlighted as an enabler for openness and building trust as it allowed for carers to get to know and feel comfortable with each other.

The breakout rooms [were] really helpful. We got to know each other and that was where we all felt safe [C15, 51, Female, White, 11 months. Exp.]

The RFP was designed for groups of 6–10 carers. However, due to recruitment issues in some sites smaller groups (4–5 carers) were accepted. The role of small group sizes in facilitating or hindering sharing depended on dynamics and carers' preferences. For most, small group size was highlighted to have facilitated sharing by allowing adequate time for each carer to share. However, for one male carer the small group size was thought to have limited adequate discussion (Table 2, quote(s) C).

#### Prior knowledge of other carers and facilitators

Some groups were mixed, consisting of carers from different fostering services without prior knowledge of each other, while in other groups most carers came from the same fostering service, knew each other and the facilitators. Although most carers from mixed groups had no strong views about group dynamics, some felt that it initially took time to build the trust required to facilitate openness, with programme structure playing a key role in creating a safe space. For single groups, although none of the interviewed carers said that having prior knowledge of the facilitators was a hindrance to their personal sharing, a few mentioned it as a potential hindrance for ‘other’ carers. However, some carers admitted that having prior knowledge of other carers influenced the depth and detail of experiences shared during the sessions (Table 2, quote(s) D).

I would probably find it easier [if I didn’t know everyone]. Because of the issues we face with [Childs name], I’m very aware that all foster carers know each other [C7, 52, Female, White, 6 yrs. Exp.]

These perspectives reveal carers having to manage their role within these activities, making decisions on what and how much to reveal in the discussion based on existing relationships.

#### The facilitator role

The authenticity and openness of facilitators, in sharing their own personal challenges (which is what the programme encourages) paved a way for carers to open up about their challenges (Table 2, quote(s) E).

The sensitivity with which the facilitators and other carers responded to the carers when they shared sensitive personal experiences, and carers’ expectations also played a key role in facilitating and hindering increased openness and further engagement. However, a few carers spoke about their experiences of sharing difficult experiences and how they perceived the feedback received from facilitators or other carers as negative leading to their disengagement (e.g., unwillingness to share again). Some carers, after sharing personal experiences, perceived offers of support from others as pity or interpreted silence as criticism (Table 2, quote(s) F). One carer shared how her expectation of getting some ideas from the group after sharing a very challenging experience, was disappointingly met with silence:

I revealed to everybody about [that] my child is violent towards me…people were sympathetic, but it was just opened up to the group. I was quite upset because it was already a difficult time and I felt that I’d really done a big thing in putting that out there and exposing that to other people in that there wasn’t any support. [C4, 56, Female, White, Foster, 9 months. Exp.]

These experiences highlight the sensitivity required of facilitators, and expectations from other carers when delivering/participating in group-based programme where vulnerable information is being shared.

Co-facilitation of the programme by a social worker and foster carer, a unique feature designed to promote epistemic trust (i.e. the willingness to accept new information as trustworthy, relevant, and generalisable), was deemed beneficial to participants. Interestingly, carers mentioned that the presence of the social worker instilled confidence, added credibility to the training and brought objectivity to discussions (Table 2, quote(s) G). Some carers, however, perceived the social worker to lack real-life experience of fostering (Table 2, quote(s) H). One carer, who described a challenging relationship with her own social worker (not the facilitator), spoke at length about the benefits of having social worker attend or deliver programmes such as RFP. This carer highlighted the programme as an opportunity for social workers to gain insight about what carers go through and therefore be better equipped to support carers (Table 2, quote(s) I).

More social workers should attend [the programme] … because people need to understand what we go through, then they might then have some empathy on us. [C24, 48, Female, Black,10 yrs. Exp.]

Most carers felt that the foster carer facilitators helped to create an environment of empathy and understanding as they could identify with participants’ experiences. The foster carer facilitator’s role was crucial for participation as they often took the lead to share their experiences, paving the way for participants to know what was expected for them as part of the activities (Table 2, quote(s) J).

### Theme 2: ‘Stopping and thinking in the moment’-carers’ understanding of programme concepts and their practical applicability

#### Understanding mentalization

The RFP is based on the key concept of mentalizing, understood to be a critical skill for building relationships, developing epistemic trust and facilitating children’s openness to listening and learning from their carers (Redfern et al., 2018). The programme therefore focusses on developing carer’s ability to understand self (their own thoughts and feelings) and focuses on developing an awareness and curiosity in what might be going on with the child’s thoughts and feelings. Mentalization has several dimensions including: (1) offline (reflection) and online (in the moment) mentalization, (2) mentalization of the self and other and (3) affective (emotions) and cognitive (thoughts) mentalization.

The concept of mentalizing is introduced explicitly early in the programme, with facilitators encouraged to explain it using everyday language and examples. Generally, most carers could relate to the concept of mentalization, emphasising that the programme simply highlights the importance of normal thought processes and paying attention to them deliberately.

It was good where they taught me about mentalization, even though you kind of do do these things without knowing that’s the label for it. But it made me do it more often [C14, 58, Female, White, 12 yr. Exp.]

However, carers’ understanding of the different dimensions of mentalization varied, determining whether and how they applied learning from the programme. Most carers who felt that they had been applying ‘reflective’ practices prior to the programme described offline mentalization (Table 2, quote(s) K). However, some carers clearly differentiated offline and online mentalization, the latter as a new practice they had adopted following the RFP (Table 2, quote(s) L).

The RFP has made me stop and think in the midst of things…as it’s actually happening, this has really helped me [C4, Age 56, Female, White, Foster, 0 yrs 9 months Exp.]

Most carers understood the concept of mentalizing the self and the other. Whilst most were familiar with mentalizing the child from previous training (albeit using different terms), learning about mentalizing the self was highlighted as new (Table 2, quote(s) M).

The side I didn’t really do much on before was…I wouldn’t think about myself in a situation where I was trying to sort out an issue that was going on with a child I was looking after. I wouldn’t stop to think, “How does this make you feel? Are you reacting the right way? [C14, Age 58, Female, White, 12 yr 8 mo Exp.]

An important aspect of mentalizing acknowledged within the programme is that *the mind is opaque*, making it difficult to know for certain what others are thinking and feeling. For this reason, the programme emphasises the importance of being curious about what could be going on for the child to build relationships. This concept, although clear and acceptable to most carers, created a stumbling block for one male carer who didn’t see the point of mentalizing, as one is unable to fully know what the other this thinking or feeling. Unsurprisingly, when asked about mentalizing this carer said (Table 2, quote(s) N):

I hated that word [mentalizing]. Because to me it’s thinking about thinking, and you can’t do that [C16, 64, Male, White, 6yrs. Exp.]

#### ‘Tools’ to facilitate mentalizing

During the sessions, several tools are introduced to help carers mentalize (see methods). Although most carers couldn’t recall the specific tool names (apart from the emotional thermometer), they could relate the concepts of what they were designed to do. Most carers felt the tools were practical and simple to apply in any given situation, without giving them rigid instructions.

I think the Reflective Fostering gives you those tools to handle those situations and feel better about you handling the situation than being told “No, this is the only way to do it” [C3, 58, Female, White, Foster, 10 Yrs. Exp.]

However, two carers felt the tools were too objective and theoretical to apply in a given situation. One of the carers, a connected carer, reported to have preferred specific instructions of what to do in a challenging situation, rather than being given tools for thinking. The second, a male carer previously mentioned to have struggled with the concept of mentalization, felt that applying the tools in a high stress situation (i.e., online mentalization) was impractical and unnatural. In addition, although this male carer could apply concepts of self-mentalization, they said that they found it easier to mentalize cognitively (thoughts) rather than affectively (feelings) (Table 2, quote(s) O).

They’re great theories, but actually the practice is what do you do. What is the best thing to do, and what is the next best thing to do? [C13, 69, Female, White, Connected, 3 yrs. Exp.]

My view is you don’t get out bed thinking, “Oh, should I use that tool”. You get out of bed and you do what you do…[these] things are subjective and objective depending on your view [C16, 64, Male, White, 6 yrs. Exp.]

### Theme 3: Practical application and perceived impact of the programme

#### Emotional regulation

One of the core mechanisms of change of the RFP is ‘carers learning to monitor their emotional temperature in care situations to better manage arousal and stress levels (Redfern et al., 2018)’. Almost all carers talked about adopting the practice of using the ‘emotional thermometer’ to monitor their emotional state and the Mind Check exercise to assess their cognitive and emotional state (Table 2, quote(s) P). Applying these practices in a challenging situation helped carers to *‘stop in the moment’*, and to *‘think before speaking’,* with better discernment of ‘when to *pick their battles’*.

It reminds you to not instantly react. Just that two minutes to think before you go in, guns blazing, and the kids know about it [C7, Age 52, Female, White, Foster 6 Yrs. 3 mo. Exp.]

Carers described the outcome of monitoring their own mental state as adopting a calm approach (as opposed to a confrontational approach) which prevented emotional outbursts and facilitated better communication. As such, some carers referred to mentalization as a *‘de-escalation tactic’*, a new way of resolving issues, one that moderates reactions (Table 2, quote(s) Q).

She’s [the child] sometimes maybe waiting for you to say something, or challenge it. And I think by not challenging it…we’ve not taken it on if you like. So, it’s saved a lot more explosive outbursts [C3, Age 58, Female, White, Foster, 10 Yrs. 2 mo. Exp.]

#### Mentalization of the self and other

Another proposed mechanism of change of the programme is *carers maintaining a sense of curiosity and an open mind about their own and the child’s mental states (reflective capacity)* (Redfern et al., 2018). Participants appeared to have explicitly taken these ideas on board and used phrases such as *‘stepping into the child’s shoes’*, *‘coming down to the child’s level’* or ‘*looking at things from the child’s point of view’* to describe mentalizing the child.

Carers gave examples of specific incidents where monitoring their own emotional state and showing curiosity about the child’s mind in the moment had moderated their actions and prevented outbursts. One carer described her thinking process during an incident where she had decided to go and look for the child when she was late in coming home. In contrast to the participant quoted earlier who struggled to utilise Reflective Fostering concepts in everyday moments (Table 2, quote(s) O), here we see a carer describing their application of concepts [shown in square brackets] to make in-the-moment decisions on the next course of action and the impact of those decisions:

But as I’ve seen her coming towards me, I stopped myself and I thought, “No, reflect, reflect, go back down there **[emotional regulation - emotional thermometer]**, think about how she's feeling now. She's obviously running. She's realised she's late. She's very anxious thinking she's going to be in a lot of trouble **[mentalizing the child - carer APP]**. We'll talk about it later [**carers decision**] … she was surprised **[child’s reaction]**, and we had a lovely meal together**[outcome].** If I’d have said something then it would have exploded and neither of us would have got anywhere. [C3, 58, Female, White, Foster, 10 Yrs. Exp.]

Most carers rightly described mentalizing as a process involving curiosity, empathy and validation. Some carers who talked about operationalizing the APP and the child MAP felt the tools enabled them to adopt a more empathetic approach. In addition, through the ‘two hands’ approach some carers better understood the need for empathy/validation while also providing boundaries. One carer described how she adopted a less punitive and disciplinarian approach, when she understood that the type and severity of punishment is not as important as the child recognising their behaviour is wrong, and that all behaviour has consequences (Table 2, quote(s) R).

Mentalizing ‘the self’, not just the child, with particular consideration of their own background and emotional state (using the carer MAP and APP) was highlighted as a learning unique to the Reflective Fostering Study (Table 2, quote(s) S).

I would get cross because my expectation of her is to be able to go off and do X, Y and Z and she doesn’t do it. But because I’d never analysed me or mentalized myself on it, it’s kind of escalating into a bit of a shouting match [C14, 58, Female, White, 12 yrs. Exp.]

Improved communication and trust between carers and the children was an outcome highlighted by some who felt the programme had improved their relationship. The carers described how monitoring their emotional arousal (emotional thermometer), choosing the right time to communicate with the child and mentalization (of self and other) had led to a more positive living environment. The end result of such processes was the development of what is termed ‘epistemic trust’ (Fonagy & Allison, 2014), that is a greater capacity to feel safe to learn from others, enabling bonding and security and safety (Table 2, quote(s) T).

#### Wider impact of the reflective fostering programme

Some carers who had observed positive outcomes, described impact beyond their relationship with the child in their current care. These carers viewed skills they had learnt from the programme as life skills, which they adopted for use in other relationships (e.g., with spouses), and contexts (e.g., work) (Table 2, quote(s) U). One carer shared how participation in the programme had given her opportunity to reflect on what went wrong in a previous placement, concluding that if she had implemented the learning from the RFP then, the placement could have potentially been saved:

I would think about ‘that would work with a previous child’. I had a teenage girl and she was with me for three years and it broke down. And I was thinking, ‘if I’d have done this earlier, I could have probably saved that placement’ [C5, 52, Female, White, 9 yrs. Exp.]

Importantly, for some carers, the programme instilled a determination in them to not give up on the children in their care and get appropriate support. For one newer carer, the programme, potentially saved a placement breakdown.

I’ve got to say that I did consider a couple of times moving him on, but I didn’t. The [Reflective Fostering Programme] really helped me. Really gave me some good tools to navigate my way through the difficulties we’ve had, and into a warm relationship. I’ve learnt it’s useful to put into practice to recognise my emotional state and to think about his thoughts and feelings as to why he is the way he’s acting. [C11, 53, Female, White, Foster, 7 months Exp.]

## Discussion

This qualitative process evaluation aimed to understand foster and kinship carers’ experiences, practical application, and perceived impact of the RFP in their relationships with children in their care.

While psychoeducation is a component of the RFP, carers’ experiences were primarily shaped by the group work component of the intervention (Redfern et al., 2018). Participant narratives regarding sharing experiences and knowledge, highlighted empathy, and validation as key aspects of a mentalizing approach which when enacted by both facilitators and carers, facilitated feelings of trust and safety, allowing carers to reflect on difficult experiences and be heard by others. In line with previous research (Logren et al., 2019), our findings suggest that, for some carers, group work served to address feelings of isolation, guilt and lack of confidence through shared learning and normalisation of experiences. Our findings resonate with previous research which highlights peer contact between foster carers to be informational (assisting with problem solving), provides empathy and counters feelings of isolation (Blythe et al., 2011; Sebba & Luke, 2013). These findings are in line with the theory of change and proposed outcomes of the RFP(Redfern et al., 2018), where the underlying assumption is that sharing similar experiences builds epistemic trust, leading to increases in carers’ ability to learn from others, and promoting a sense of confidence and competence around parenting skills.

The sharing of challenging experiences related to children in their care was also identified as an important aspect of the programme, allowing both facilitators and participants to actively model and practise mentalizing, using the tools introduced. Feelings of trust and safety experienced in the group emerged as a key aspect that enabled carers to share challenging experiences. Whilst the programme structure and activities (e.g. Mind Check) played an important role in facilitating feelings of trust and safety, our findings give some insight about the conditions under which trust and safety is experienced and what enables them to manifest.

Firstly, the findings reveal the importance of group composition (i.e. previous knowledge of other carers and facilitators) in facilitating/hindering trust and disclosure of experiences/vulnerabilities. Perspectives shared in this study reveal carers managing their role within the group activities by considering what would be at stake for both the child(ren) in their care and their credibility and accountability, to inform decisions regarding whether and how much to share. This finding aligns with previous research investigating self-disclosure in group counselling, which found that in sharing of experiences, individuals take into account the sociocultural values that are linked to the objects in discussion and the dilemma of epistemic access to experience (Logren et al., 2019). It is also important to note that two of the three male carers, who participated in our study expressed negative views regarding sharing of challenging experiences. Although these participants had different reasons for holding such views (i.e., finding sharing negative experiences de-motivating and small group size limiting in-depth discussions), our findings may suggest that some male carers might struggle with this dynamic, and require a different kind of approach. Men’s underutilisation of psychological interventions is well documented (Cochran, 2005). Previous research has also suggested that men can appear to be more sceptical about disclosure, and seem evasive and uncertain about emotional expression (Cochran, 2005). Our findings might suggest the need for empirically tailored interventions to enhance uptake and engagement in men, along with formal evaluations to advance the evidence base (Seidler et al., 2018).

Secondly, our findings suggest that epistemic trust and programme participation can be facilitated by facilitator disclosures of difficult personal experiences as carers themselves. It appeared that disclosure of difficult experiences by social workers, although highlighted by some participants as out of the norm, facilitated epistemic trust by balancing power dynamics. In addition, personal experiences shared by foster carer facilitators, which were highly relatable to participants, appeared to be vital for demonstrating intervention activities including practical application of psychoeducation. This finding underscores the importance of co-facilitation of the programme by a foster carer and social worker, resonating with previous qualitative evaluations of the RFP (Midgley et al., 2021b) where participants found the inclusion of a foster carer as a co-facilitator to enable the development of a safe, non-judgmental space for open communication. Our study adds to these findings by identifying the perceived importance of including a social worker (i.e., credibility) and by highlighting important considerations for their facilitation (i.e. adopting a different way of relating to carers to their usual professional stance).

Thirdly, our findings highlight the critical communication skills facilitators need to deploy to maintain trust following carers’ disclosures of difficult experiences or displays of vulnerability. For example, where carers perceived silence as criticism, and offers of support as pity, this study makes clear the importance of facilitators affirming the carer’s experience and being careful not to expose them to perceived criticism or pity. Previous research acknowledges the delicacy of either affiliating or disaffiliating with experiences when responding to self-disclosure whilst maintaining positive relationships (Logren et al., 2019). This previous research suggests presenting a second self-disclosure based on the speaker’s own experiences as an effective way to challenge the other or affirm disclosures in a group whilst maintaining relationships (Logren et al., 2019; Pino, 2017). Whilst our findings reveal negative experiences for a few carers regarding responses received after sharing difficult experiences, they provide valuable insights into the dynamics of running the sessions, highlighting the importance of how facilitators respond to ‘critical moments’ in programme delivery, that is, when carers share difficult experiences. To do this clearly requires sophisticated communication skills. Therefore, the negative experiences reported in this paper provide clues for how to refine the training of facilitators and could be helpful for informing where delivery of the programme might be adapted to facilitate sharing from carers.

Our examination of carers’ narratives of practical application of learning from the Reflective Fostering Programme, suggest the programme can successfully facilitate the practical implementation of self-mentalizing, leading to greater self-regulation and improvements in the carer-child relationship. Moreover, our findings suggest that self-regulation enacted through monitoring emotional arousal, and mentalizing the self (and other), was felt to help regulate carers’ behaviour (e.g., carers choosing to avoid immediate confrontation of issues) leading to the prevention of outbursts and better communication between the child and the carer. These findings resonate with theory underpinning the RFP which associates higher reflective functioning in caregivers with a higher tolerance to distress in their children (Rutherford et al., 2013), the ability to manage stressful situations (Borelli et al., 2016), and better communication with their children (Rostad & Whitaker, 2016). Our findings, highlighting the central tenet of the RFP (i.e., focus on carers' self-regulation and self-mentalizing), differ to those reported in qualitative evaluation of other trauma and attachment informed programmes (e.g., Fostering Connections) (Lotty, Dunn-Galvin, & Bantry-White, 2020), where carers report more implicit outcomes (e.g. changes in perceptions about the children’s emotional and behavioural difficulties) (Lotty, Bantry-White, & Dunn-Galvin, 2020).

Finally, our findings, providing some insight into key aspects that facilitate the application of learning from the Reflective Fostering Programme, reveal that carers' understanding of the concepts of the programme (i.e., mentalization), their limitation (i.e., that mind is opaque) and how they differ or relate to other known concepts (e.g., online and offline reflection), are important to their application in relationships with children in their care.

## Strengths and limitation

This study examined carers' experiences, understanding and application of the RFP in depth, engaged in as part of a large-scale randomised clinical trial. Previous qualitative research on the RFP has provided feedback on acceptability by exploring relevance of the programme and carers' views on structure, content and co-delivery of the Programme (Midgley et al., 2019; Midgley et al., 2021). The current evaluation adds to this body of research by beginning to understand, from carers’ perspectives, how mechanisms of change are enacted within sessions and beyond to have an impact on the carer-child relationship. Our findings therefore provide valuable insight on how to refine and adapt the delivery of the programme including training for facilitators to enhance practical application.

## Conclusion

This study suggests successful enactment of key aspects (i.e. self-regulation and self-mentalizing) of the RFP by most carers. Reported benefits of the programme included enhanced capacity to cope with stressful situations, prevention of outbursts and improved communication, align with the programme model and underpinning attachment and mentalization theory. Our findings highlight the sharing of challenging experiences, as a key aspect of the programme that enabled active modelling and practising of mentalizing. They also highlight group composition, facilitator disclosures of difficult experiences and sensitive communication skills as key to creating an atmosphere of trust which in turn facilitates carer disclosures. Further research is needed to understand the role of gender, ethnicity and carer type in programme engagement and enactment.

## Supplemental Material

Supplemental Material - A qualitative evaluation of the reflective fostering programme – examining foster and kinship carers’ experiences, practical application, and perceived impact Supplemental Material for A qualitative evaluation of the reflective fostering programme – examining foster and kinship carers’ experiences, practical application, and perceived impact by Thando Katangwe-Chigamba, Jamie Murdoch, Karen Irvine, Sheila Redfern and Nick Midgley in Clinical Child Psychology and Psychiatry.
