# Supplementary material for: A qualitative evaluation of the reflective fostering programme – examining foster and kinship carers’ experiences, practical application, and perceived impact
Source: Clin Child Psychol Psychiatry. 2025 Feb 19;30(3):588–610. doi: 10.1177/13591045251321032 (PMC12179401; doi:10.1177/13591045251321032)
Supplement: Supplemental Material - A qualitative evaluation of the reflective fostering programme – examining foster and kinship carers’ experiences, practical application, and perceived impact [file sj-pdf-1-ccp-10.1177_13591045251321032.pdf]

# Reflective Fostering Programme

## GUIDE FOR INDIVIDUAL INTERVIEWS (CARERS)

**1. Welcome**

*Introduce yourself and thank the participant for agreeing to take part in the interview.*

**2. Introduction**

As you know, the aim of the interview today is to find out about your experiences of taking part in the Reflective Fostering Programme. We're interested in hearing about your personal views and experiences - there are <u>no right or wrong answers</u>. I have a list of suggested questions to remind me of the kind of things we might want to talk about but exactly what we cover is up to you. Feel free to go into as much detail as you feel comfortable with. If you don't feel comfortable talking about something then that's absolutely fine, just let me know – we can change the topic, take a break or stop the discussion at any time. Do you have any questions before we get started?"

*Answer any questions the participant has. Check that they are comfortable and let them know before turning on the recorder.*

**3. Questions**

*Note: The questions that follow are only a guide. Do not follow these prescriptively but be led by the interests and concerns of the participant. Use follow-up questions to clarify the participant's response or elicit additional detail when needed. Follow-up questions might include:*

- *Are you able to tell me more about that?*
- *Can you give an example of a time when that happened/ when you felt like that?*
- *What did you think about that?*
- *What happened (next)?*
- *Do you mean …?*

**4. Opening question**

- Please begin by telling me about your experience of being a foster carer/kinship carer for the child(ren) in your current placement.

  *Prompt for:*
  - *Whether their experience has (or hasn't changed) changed over the past 12 months.*
  - *Whether their relationship with the child(ren) in their care has changed in the past 12 months.*

- *The reasons for any changes in their experience (if any).*
- *Whether they can attribute any changes in their experience/relationship with child(ren) to the Reflective Fostering intervention or usual support.*
- *Whether they can attribute any changes in their experience/relationship with child(ren) to the Pandemic e.g., lockdown and school restrictions.*

**5. Additional questions (RFP only)**

- Can you tell me about your experience of being part of the Reflective Fostering Programme?

  *Prompt for:*
  - *Was there a particular session or a particular moment that sticks in your mind? Tell me about it*
  - *Were there any specific ideas/tools that you found particularly useful or difficult and why?*
  - *How useful was the emotional thermometer/Carer APP/MAP/Two hands approach for understanding your relationship with the child? Can you give me an example of when you used the xxxx with your child?*
  - *How useful was mentalisation in your relationship with the child in your care? Can you think of an example?*

- Can you think of a time at home where you feel you made use of something that you learnt from the Programme/training?

  *Prompt for:*
  - *Please tell me what happened?*
  - *What was it about the programme that you used there?*
  - *How might this have gone differently before you attended the Programme/training?*
  - *Is there a time that you have been able to use what you've learnt from the Programme on other children in your care? (if have more than one placement or have own children)*
  - *Is there something that you learnt from the programme that you feel you are using 12 months on?*

- Can you tell me about how the sessions were run? What was helpful/less helpful?

  *Prompt for:*
  - *How did you find doing the activities with other carers? How do you think other carers found the sessions?*
  - *Which activities worked/didn't work well? Why was that?*
  - *What was you experience in sharing and hearing different carer experiences?*
  - *Some of the sessions asked you to share difficult experiences you have had with the child in your care. How did you find that?*
  - *What are your thoughts on having the sessions led by a social worker and a foster carer?*
  - *What was the dynamic between the foster carer and social worker leading the session and did this impact your learning?*

- What type of support have you received from your social worker and the child's social worker in the previous 12 months?
  *Prompt for:*
  - *What was did you find helpful/not helpful?*
  - *Did participation in RFP influence your relationship with your/your child's social worker at all?*

- What other forms of support have you received in the previous 12 months?
  *Prompt for:*
  - *What support have you received from the child's school? Did you find it helpful/unhelpful?*
  - *Has there been any referrals to specialist support e.g. CAMHS? Did you find it helpful/unhelpful?*
  - *Are there any informal support networks that you have found helpful e.g. family, friends and other foster carers*

**6. *Additional questions***

- Can you tell me about your experience of taking part in any other training provided by your Local Authority?

  *Prompt for:*
  - *What made you decide to take part in that training?*
  - *What was the training about?*
  - *What was helpful/less helpful about the training?*
  - *Were there any specific ideas/tools that you found particularly useful? Which ones were less useful?*
  - *Is there a particular experience in that training that sticks in your mind? Tell me about it*
  - *Did the Reflective Fostering Programme overlap with any of this training or was it different? If so, in what way?*

- Can you tell me how the training was run?

  *Prompt for:*
  - *How long and often was the training?*
  - *Was it delivered online or face to face?*
  - *Did the training involve activities with other carers? If yes, in your view which activities worked/didn't work well? Why is that?*

- Can you think of a time at home where you feel you made use of something that you learnt from the training?

  *Prompt for:*
  - *Please tell me what happened?*
  - *What was it about the training that you used there?*
  - *How might this have gone differently before you attended training?*
  - *Is there something that you learnt from the training that you feel you are using now?*

**7. Additional questions (Both arms)**

- What have your wider experiences of taking part in the research been?

  *Prompt for:*
  - *How did you hear about the study?*
  - *What influenced your decision to take part?*
  - *How did you find communication about the study e.g. what its about, which arm you were allocated in and completion of measures*
  - *What are you experiences with completing measures of the study?*
  - *What do you hope would result from being part of a research study of this sort?*
  - *Would you recommend being part of the research to others?*
  - *Anything that could be done differently.*

- Do you feel that your role as a carer has changed over the course of the study? [Or of being a parent]?

**8. Close**

Before we finish, is there anything else about your experiences of taking part in the Reflective Fostering Programme/study that would be useful for us to know?

*Thank the participant for their time and for sharing their experiences and opinions.*

*Explain what will happen to the interview data and how the findings will be used.*
